# 第六届中国肺癌南北高峰论坛在北京全国政协礼堂隆重召开

**DOI:** 10.3779/j.issn.1009-3419.2013.12.08

**Published:** 2013-12-20

**Authors:** 修益 支

**Affiliations:** 100053 北京，首都医科大学肺癌诊疗中心，首都医科大学宣武医院胸外科 Beijing Lung Cancer Center, Department of Toracic Surgery, Beijing Xuanwu Hospital, Capital Medical University, Beijing 100053, China

2013年11月16日，由中国癌症基金会、中国抗癌协会、首都医科大学和中国健康促进联盟共同主办、首都医科大学肺癌诊疗中心、中国医科院肿瘤医院和首都医科大学宣武医院承办的“第六届中国肺癌南北高峰论坛暨2013年全国肺癌诊疗新技术新进展学习班”在北京全国政协礼堂隆重召开。全国人大常委会副委员长、中华医学会会长陈竺院士和全国政协副主席、中国健康促进联盟名誉主席韩启德院士担任论坛名誉主席，原全国人大副委员长、中国癌症基金会主席、论坛名誉主席何鲁丽女士出席了开幕式。本届论坛主题：关注控烟与肺癌早诊早治、推动肺癌规范化个体化诊疗。

上午8时，第六届中国肺癌南北高峰论坛开幕式在中国癌症基金会控烟与肺癌防治工作部主任、首都医科大学肺癌诊疗中心主任、宣武医院胸外科主任支修益教授和中国抗癌协会肿瘤临床化疗专业委员会主任委员、中国医学科学院肿瘤医院副院长石远凯教授共同主持下开始。论坛主席、中国癌症基金会理事长彭玉女士发表了热情洋溢的开幕词，中国健康促进联盟常务副主席蔡纪明、中国医师协会胸外科医师分会会长王天佑、国家癌症中心副主任董碧莎、中国农工民主党中央副主席何维和国家卫生计生委宣传司副司长姚洪文先后致辞讲话。世界卫生组织驻华控烟官员Angela Pratt和美国国立癌症研究所全球健康中心东亚处主任Ann Chao应邀出席，并在开幕式上发言中祝贺第六届中国肺癌南北高峰论坛召开并传递国际社会的控烟与肺癌防治的最新信息。论坛主席、首都医科大学校长吕兆丰教授发来书面致辞，论坛主席、中国健康促进联盟主席、中华预防医学会会长王陇德院士题写了“控制烟草危害，维护国民健康”的贺词。我国胸外科老前辈、96岁高龄的黄国俊教授、中国健康促进联盟成员单位负责人、我国控烟与肺癌防治领域著名专家学者以及首都30余家新闻媒体共400余人参加了本届高峰肺癌论坛。

第六届中国肺癌南北高峰论坛的议题包括：我国控烟与肺癌防治策略、肺癌筛查与早诊早治项目、早期肺癌诊疗策略和微创技术应用、肺癌临床诊疗规范解析、转化医学研究成果应用与创新、基于基因检测指导下的肺癌个体化治疗、改善晚期肺癌患者生活质量，以及有关抗癌新药和戒烟药物进入国家医保目录建议等诸多方面。中国科学院肿瘤医院副院长王绿化、中国医药工业研究开发促进协会执行会长宋瑞霖和中国医师协会胸外科医师分会副会长叶玉坤教授主持上午的大会，我国控烟与肺癌防治策略7位著名专家在上午的大会上作了主题发言，分别是：中国抗癌协会副理事长于金明院士（肺癌放射治疗新进展），中国控制吸烟协会常务副会长兼秘书长许桂华（低焦油不等于低危害），中国癌症基金会常务副理事长兼秘书长赵平（全球肺癌流行趋势与防控策略），国家卫生计生委疾控局副局长王斌（中国癌症防控策略与癌症早诊早治工作），中国医科院肿瘤医院教授孙燕院士（肺癌临床研究进展2012-2013），中国医科院肿瘤研究所研究员程书钧院士（肺癌防治战略前移），原中国疾控中心副主任兼控烟办公室主任杨功焕（国际和中国控烟进展）。

下午分别设立了中国胸外科主任肺癌高端论坛曁中国胸外科肺癌协作组专题研讨会和肺癌内科治疗新进展高端论坛两个分会场，中国胸外科肺癌协作组副组长高文和何建行、中华胸心血管外科学会肺癌学组副组长刘伦旭，北京医学会胸外科分会刘德若、李简和杨跃主持了“中国胸外科主任肺癌高端论坛”，中国癌症基金会控烟与肺癌防治工作部副主任邹小农（中国控烟与肺癌防治），中国医学科学院肿瘤医院影像诊断科副主任吴宁（早期肺癌筛查），中国抗癌协会临床肿瘤协作中心主任委员、中国胸部肿瘤协作组组长吴一龙（肺癌综合治疗和临床试验），中华胸心血管外科学会肺癌学组组长、中国胸外科肺癌协作组组长支修益（早期肺癌外科诊疗新策略），中国抗癌协会肺癌专业委员主任委员王长利（2013 WCLC-NSCLC治疗新进展），中山大学肿瘤医院胸外科主任张兰军（NSCLC综合治疗策略：胸外科视角），首都医科大学肺癌诊疗中心副主任李辉（胸腔镜技术在早期肺癌外科治疗中的地位）、天津市肺癌研究所所长周清华（Ⅲa-N2局部晚期非小细胞肺癌外科诊疗新策略）和中华胸心血管外科学会肺癌学组副组长、广州医科大学第一附属医院院长何建行（基于基因指导下的NSCLC术后个体化辅助治疗）作了专题演讲。

中国肺癌防治联盟主席白春学、中国医科院肿瘤医院肿瘤内科冯奉仪、北京大学肿瘤医院肺癌内科主任王洁和解放军总医院第一附属医院肿瘤一科主任肖文华主持了“肺癌内科治疗新进展高端论坛”，中国医学科学院肿瘤医院副院长石远凯[《肺癌骨转移专家共识》（2014年版）解析]，中国疾病预防控制中心控烟办公室研究员杨杰（国内外全面无烟环境立法和执法进展），上海市胸科医院副院长韩宝惠（NSCLC二线治疗新策略），中华医学会呼吸分会副主任委员白春学（如何进行肺癌的早期诊断），首都医科大学肿瘤学系肺癌学组副组长张树才（晚期非小细胞肺癌治疗的思考），全军肿瘤专业委员会副主任委员焦顺昌（肺癌的免疫治疗进展），中国医学科学院肿瘤医院检验科副主任韩晓红（肺癌靶点检测的技术），浙江大学附属第一医院胸部肿瘤科主任赵琼（晚期肺鳞癌治疗的挑战与机遇），山东省肿瘤医院内科主任郭其森（2013 ESMO NSCLC治疗亮点），中国老年肿瘤学会肺癌专委会主任委员程刚（晚期非小细胞肺癌研究进展与思考），天津医科大学肿瘤医院肿瘤内科主任李凯（抗血管生成治疗：患者如何从中受益？）作了主题发言。

在第六届中国肺癌南北高峰论坛期间，美国礼来公司和英国阿斯利康公司在论坛期间分别举办了第15届世界肺癌大会会后专题卫星会，介绍了11月初在澳大利亚悉尼举行的第15届世界肺癌大会相关最新信息，特别是非小细胞肺癌术前新辅助化疗、术后辅助治疗和分子靶向治疗的研究进展。法国赛诺菲公司举办了“聚焦前沿-赛诺菲肺癌专题论坛”。同历届中国肺癌南北高峰论坛一样，针对广大中青年胸外科和临床肿瘤医师的国家级继续教育项目—“2013年全国肺癌诊疗新技术新进展学习班”同期举行，来自全国各地中青年胸外科和肿瘤内科医师60余人参加了为期三天的学习班。

每年11月份是国际肺癌关注月，第15届世界肺癌大会11月初刚刚在澳大利亚悉尼落下帷幕。第六届中国肺癌南北高峰论坛在积极倡导“控烟与肺癌早诊早治、推动肺癌规范化个体化诊疗”论坛主题的同时，及时分享第十五届世界肺癌大会有关全球控烟策略、早期肺癌筛查和肺癌诊疗领域进展的最新信息，并推出了在孙燕院士主持下中国专家组完成的《肺癌骨转移诊治专家共识》（2014年版）。论坛期间，支修益和石远凯教授主持召开了国家卫生计生委《原发性肺癌诊疗规范》（2014年版）专家组会议，讨论更新《原发性肺癌诊疗规范》（2011年版）的相关内容，国家卫生计生委医政管理局主管领导出席会议并对规范更新工作作了重要指示。

本届中国肺癌南北高峰论坛得到了协办单位-中华医学会胸心血管外科学会肺癌学组、中国抗癌协会肺癌专业委员会、中国抗癌协会化疗专业委员会、中国胸外科肺癌协作组CTLCG、中国肺癌防治联盟CAALC、中国医药工业研究开发促进协会、中国农工民主党医疗卫生委员会和北京医学会胸外科分会的大力支持，

中国肺癌南北高峰论坛汇集了国内控烟与肺癌防治领域的顶尖专家与学者，为共同协商和探讨我国控烟与肺癌防治策略搭建了一个互相交流与合作的平台，作为我国控烟与肺癌防治领域的品牌论坛，为推动我国控烟与肺癌防治事业的进步与发展做出了贡献。

